# *Coxiella burnetii* infection in the lumbar vertebra: a rare case report and review of literature

**DOI:** 10.3389/fmed.2025.1618670

**Published:** 2025-08-01

**Authors:** Xingguo Tan, Feng Li, Tao Zhang, Mingjia Song, Lian Zhang, Yuan Xing, Yizhe Wang, Long Chen, Dashuai Huang, Yanpeng Lu, Songkai Li

**Affiliations:** ^1^Department of Spinal Surgery, the 940th Hospital of the Joint Logistic Support Force of the Chinese People’s Liberation Army, Lanzhou, China; ^2^First Clinical Medical School, Gansu University of Chinese Medicine, Lanzhou, China; ^3^Department of Orthopedics, the 943rd Hospital of Joint Logistic Support Force of Chinese People’s Liberation Army, Wuwei, China; ^4^Department of Clinical Laboratory, the 940th Hospital of the Joint Logistic Support Force of the Chinese People’s Liberation Army, Lanzhou, China

**Keywords:** *Coxiella burnetii*, Q fever, infection, lumbar, case report, review

## Abstract

*Coxiella burnetii* is a bacterial pathogen of Q fever. *Coxiella burnetii* infection in the lumbar vertebra is a rare form of chronic Q fever, which poses significant obstacles in both diagnostic processes and therapeutic interventions. A 57-year-old male patient with a previous diagnosis of lumbar infection with unknown pathogen at another institution was admitted for treatment. At our institution, the patient underwent surgical interventions, including decompression through total laminectomy, lesion excision, intervertebral bone graft fusion, and fixation. Intraoperative pathological samples were analyzed using a specific multiplex quantitative polymerase chain reaction (qPCR) pathogenic microorganism detection, confirming the presence of *Coxiella burnetii*. Postoperatively, the patient received long-term antibiotic therapy by oral doxycycline and ciprofloxacin for a duration plan of 18 months. At the 6-month post-operative evaluation, the patient exhibited complete resolution of clinical symptoms, and imaging results revealed no evidence of infection recurrence, suggesting a clinical cure. The combination of decompression through total laminectomy, lesion excision, intervertebral bone graft fusion, and fixation alongside oral doxycycline and ciprofloxacin treatment has been demonstrated to be an effective therapeutic strategy for managing *Coxiella burnetii* infection in the lumbar vertebra.

## Introduction

*Coxiella burnetii* is a gram-negative, fastidious, obligate intracellular microorganism classified within the family Legionellaceae (1–3). The zoonotic disease it causes, Q fever, was first identified in abattoir workers during the 1930s and derives its name from its initial “query” status due to unknown etiology (4, 5). Domesticated and wild ruminants serve as the primary reservoir, and the most common transmission mode for humans and animals is inhalation of contaminated aerosols (6).

Q fever exists in both acute and chronic forms. The incubation period may extend up to 2–4 weeks, with infections presenting in either form. Acute Q fever is typically characterized by fever, fatigue, and myalgia, sometimes accompanied by pulmonary involvement and liver impairment (7, 8). Notably, approximately 60% of acute infections are mild or asymptomatic, generally resolving spontaneously within 2 weeks, with a favorable prognosis. Less than 6% of cases progress to chronic infection, which can occur more than 6 months post-exposure. Chronic Q fever primarily involves the endocardium, cardiovascular, lymphatic, and musculoskeletal systems. Chronic Q fever carries a poor prognosis, associated with significant risks of complications (61%) and a disease-related fatality rate of 25% (9). According to prior research, the global annual incidence rate of Q fever is approximately 0.38 cases per million (10, 11), with musculoskeletal involvement occurring in less than 2% of all documented cases (12). Spinal *Coxiella burnetii* infections are exceedingly rare, and there is currently no standardized treatment protocol (13, 14).

In April 2024, a patient with lumbar infection of unknown etiology was admitted to the Department of Spine Surgery at the 940th Hospital of the Joint Logistics Support Force of the Chinese People’s Liberation Army. The diagnosis of lumbar *Coxiella burnetii* infection was confirmed via a specific multiplex quantitative polymerase chain reaction (qPCR) pathogenic microorganism detection. Due to the patient’s severe lower back pain and neurological symptoms affecting the lower extremities, surgical intervention consisting of decompression through total laminectomy, lesion excision, intervertebral bone graft fusion, and fixation was performed. Following the surgery, a duration plan of 18-month course of oral doxycycline and ciprofloxacin was prescribed as anti-infective therapy. At 6 months after surgery, the patient’s clinical symptoms were completely relieved, and postoperative imaging indicated no signs of recurrent infection, suggesting a clinical cure. Informed consent was obtained from the patient for the inclusion of their medical information in this article.

## Case presentation

**FIGURE 1:**
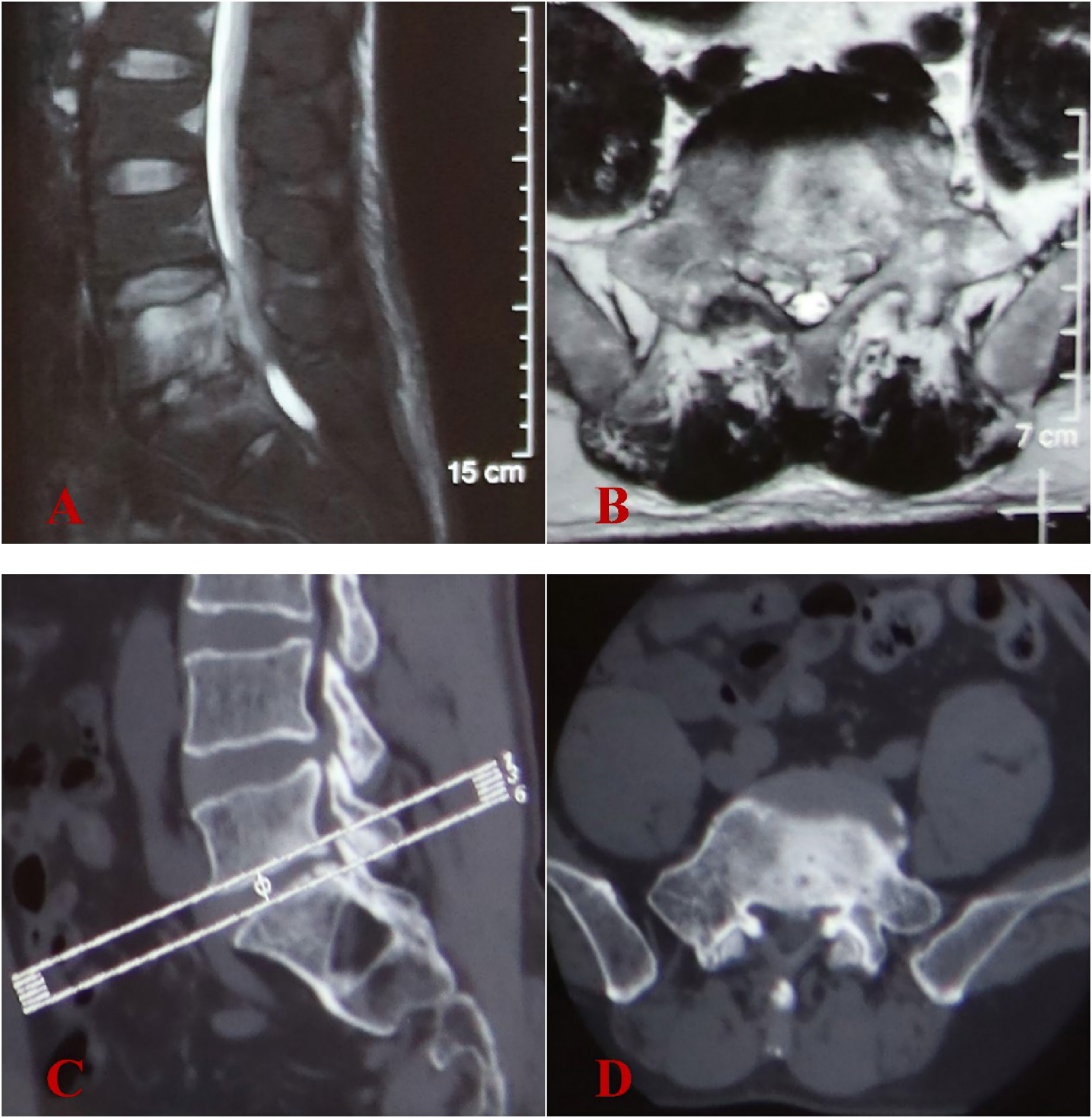
Initial image findings at another hospital in December 2023. **(A,B)** Lumbar MRI showed abnormal signals in the L5-S1 intervertebral space and L5 vertebral body, with intraspinal abscess formation at L5 vertebral level. **(C,D)** Lumbar CT scans revealed no evidence of significant vertebral bone destruction.

A 57-year-old male patient living in a rural setting with a history of sheep farming and repeated exposure to ticks, sought medical care at another hospital on December 20, 2023. He reported a 2-month history of worsening lower back pain, which had recently been accompanied by bilateral pain in the lower limbs for the preceding 2 weeks. Additionally, the patient experienced night sweats and night pain. Initial lumbar magnetic resonance imaging (MRI) indicated abnormal signals in the L5/S1 intervertebral space, suggesting the presence of an infection (Figures 1A, B). However, lumbar computed tomography (CT) scans revealed no significant vertebral bone destruction (Figures 1C, D). The patient underwent percutaneous endoscopic transforaminal lumbar discectomy and decompression of lumbar nerve root. The culture results of intraoperative specimens from L5/S1 intervertebral space were negative. Histopathological examination revealed chronic granulomatous inflammation. Despite receiving 5 months of empirical anti-tuberculosis therapy following surgery, there was no marked improvement in his lower back pain nor in the bilateral lower limb pain.

**FIGURE 2:**
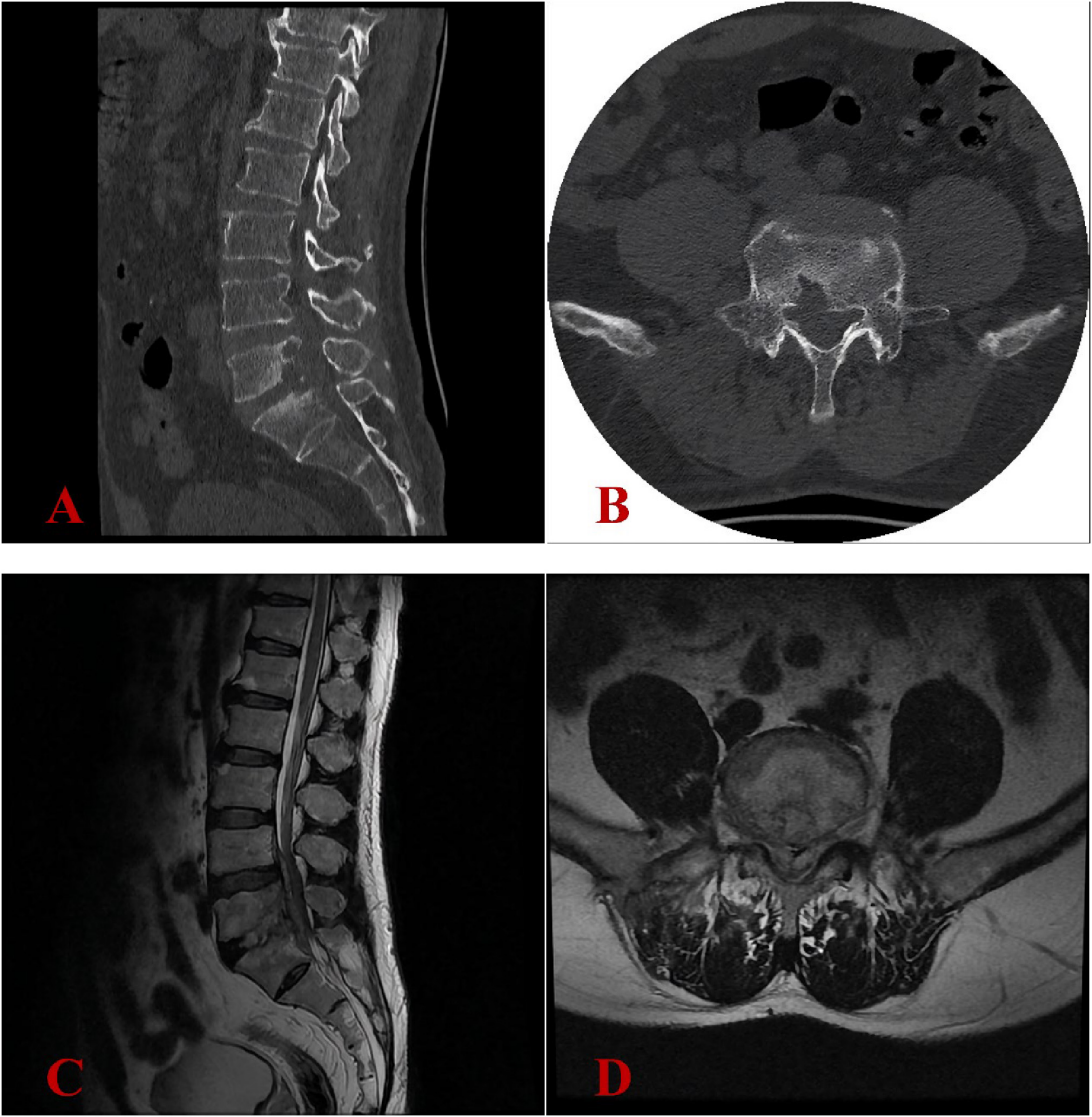
Image findings at our hospital in April 2024 revealed progression of the pathological process. **(A,B)** Lumbar CT scans revealed severe bone destruction at the posterior margin of the L5 vertebral body and the superior margin of the S1 vertebral body. **(C,D)** Lumbar MRI confirmed chronic osteomyelitis at the L5-S1 level with expansion of epidural abscesses (previously noted in December 2023) and multiple small epidural phlegmons, resulting in high-grade spinal stenosis with spinal cord compression.

The patient was admitted to our hospital on April 25, 2024. The primary clinical findings included positive tenderness and percussion pain localized to the L4-S1 region. Lumbar mobility was limited, and decreased skin sensitivity was noted in the bilateral buttocks, posterolateral thighs, and posterolateral calves. The Visual Analogue Scale (VAS) score (15) for pain intensity in the lumbar region, graded on a 0–10 scale, demonstrated a moderate-severe level of 6/10. The Oswestry Disability Index (ODI) (16), which quantifies disability in chronic low back pain patients, showed a moderate-severe impairment score of 62/100. Laboratory evaluations, including a complete blood count, liver and kidney function tests, coagulation profile, procalcitonin (PCT), erythrocyte sedimentation rate (ESR), and C-reactive protein (CRP), were all within normal limits. The tuberculosis-specific cellular immune response assay, Rose Bengal plate agglutination test, cryptococcal antigen (CrAg) detection test, and Aspergillus galactomannan (AG) immunoassay were all negative. Additionally, blood and urine cultures failed to isolate any pathogenic organisms. While serological tests would indeed provide valuable information about Q fever stage, this capability was unavailable at our institution during the diagnostic workup. Lumbar CT scans showed severe bone destruction at the posterior margin of the L5 vertebral body and the superior margin of the S1 vertebral body (Figures 2A, B). Lumbar MRI revealed abnormal signals within the spinal canal from the lower margin of L4 to the S1 vertebral plane, the L5-S1 intervertebral space, and the right intervertebral foramen, as well as abnormal signals in the L5 and S1 vertebral bodies, further supporting the diagnosis of infection with abscess formation (Figures 2C, D). Additional assessments revealed a hepatic hemangioma and splenomegaly on abdominal ultrasound, while electrocardiogram, chest radiograph, cardiac ultrasound, and bilateral lower limb vascular ultrasound showed no significant abnormalities.

### Operative procedure

Under general anesthesia, the patient was placed in the prone position. A midline incision of 8 cm length was made in the lumbosacral region. The incision extended sequentially through the skin, subcutaneous tissue, and lumbodorsal fascia. Bilateral laminae and facet joints of L5-S1 were exposed via subperiosteal dissection. Pedicle screws were inserted into the pedicles of L5 and S1 vertebrae. Total laminectomy was performed at the L5-S1 interspace. Intraoperative exploration revealed extensive fibrotic scar tissue and adhesions within the right intervertebral foramen. Extensive purulent exudate and granulation tissue were noted in the right epidural space, extending from the lower margin of the L4 vertebra to the S1 vertebra. The L5-S1 intervertebral disk exhibited herniation into the spinal canal with granulomatous changes and contained purulent fluid. Decompression was performed at the L5-S1 interspace. The thickened ligamentum flavum was removed, followed by meticulous excision of granulation tissue within the spinal canal. The L5-S1 intervertebral disk was completely resected and curettage. Posterior decompression confirmed complete release of the bilateral L5 and S1 nerve roots. The harvested spinous processes, laminae, and facet joints were pulverized and combined with allogeneic bone, rifampicin (2 g), isoniazid (0.1 g), and vancomycin (0.5 g). This mixture was then implanted into the L5-S1 intervertebral space. Fluoroscopic guidance ensured accurate placement of the internal fixation system and graft material. After achieving hemostasis, the surgical field was irrigated thoroughly with normal saline. A drainage tube was placed, and the incision was closed in layers.

### Postoperative management

**FIGURE 3:**
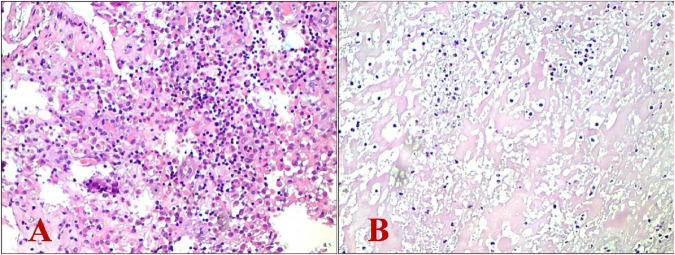
Histopathological examination revealed chronic suppurative inflammation with necrosis alongside the proliferation of inflammatory granulation tissue. **(A)** Hematoxylin-eosin stain (magnification: × 100); **(B)** Hematoxylin-eosin stain (magnification: × 200).

**FIGURE 4:**
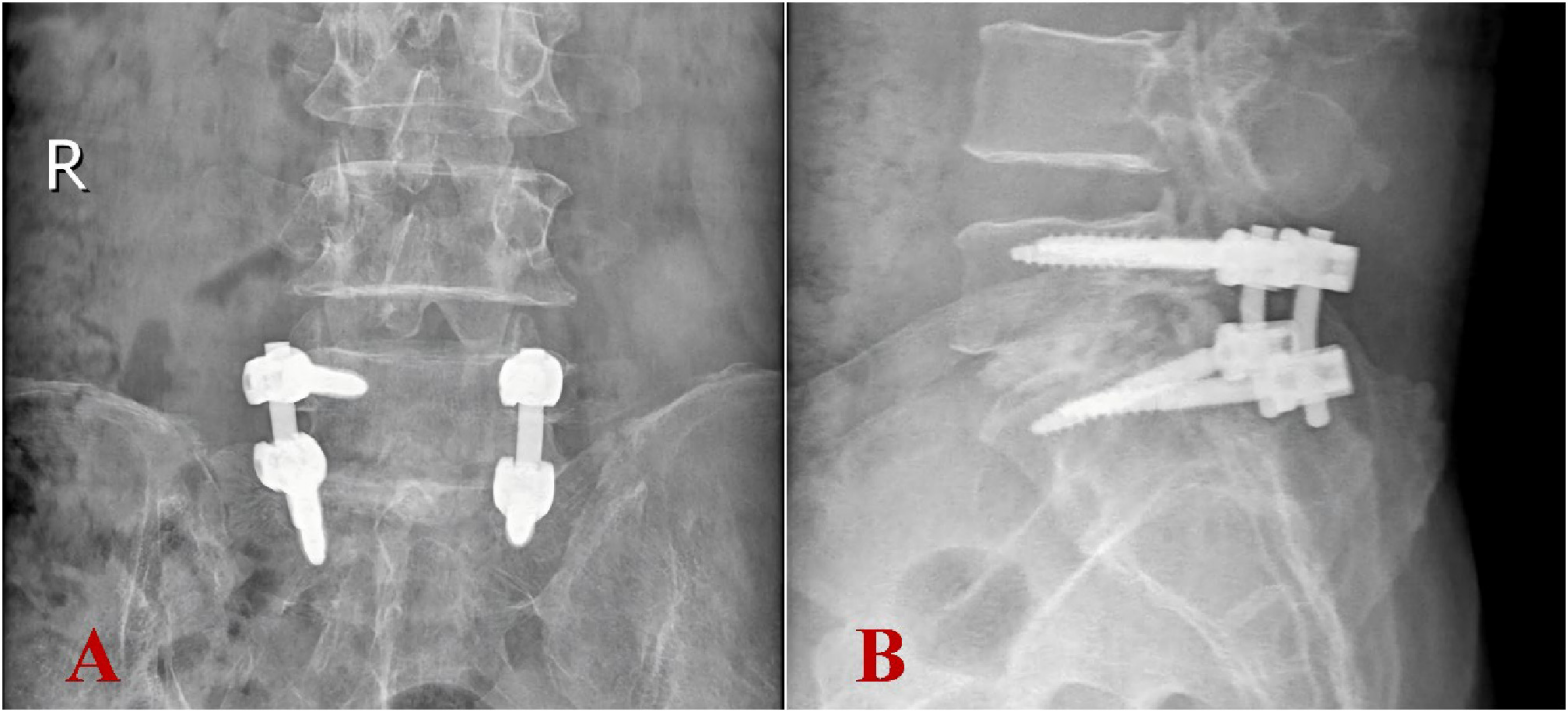
Postoperative imaging revealed appropriate positioning of internal fixation devices and bone grafts. **(A)** Anteroposterior view; **(B)** lateral view.

On the day following surgery, the patient was instructed to wear a waist girdle while ambulating. Cefuroxime sodium was administered perioperatively for 24 h to prevent infections. Intraoperative pathological specimens (granuloma tissues) were analyzed using a specific multiplex qPCR assay (Hebei Qianye Biotechnology Co., China) targeting over 100 clinically relevant pathogens, including 41 bacterial species, 25 viral species, 19 fungal species, 11 atypical pathogens (e.g., *Mycoplasmas spp., Rickettsia spp., Spirochetes*, and *Coxiella burnetii*), and 8 drug-resistance genes. The *Coxiella burnetii*-specific primers (targeting the *CBU_0340* gene; F: 5′-TTACCCAAGGCCTTTGAGATAGAAT-3′; R: 5′-CAAAACGCTCGATGGAACTGATAT-3′) demonstrated positive amplification with a Ct value of 29.24, while all other bacterial targets showed no detectable signals. On the day of definite diagnosis, antibiotic treatment was initiated with oral doxycycline at a dose of 0.2 g once daily and oral ciprofloxacin at 1 g once daily. As expected, the culture results of intraoperative specimens from L5/S1 intervertebral space were negative. Postoperative histopathological examination showed chronic suppurative inflammation with necrosis alongside the proliferation of inflammatory granulation tissue. Additionally, no typical tuberculosis imaging characteristics were noted (Figures 3A, B). Postoperative imaging showed that the internal fixation and bone graft positions were satisfactory (Figures 4A, B).

### Follow-up results

**FIGURE 5:**
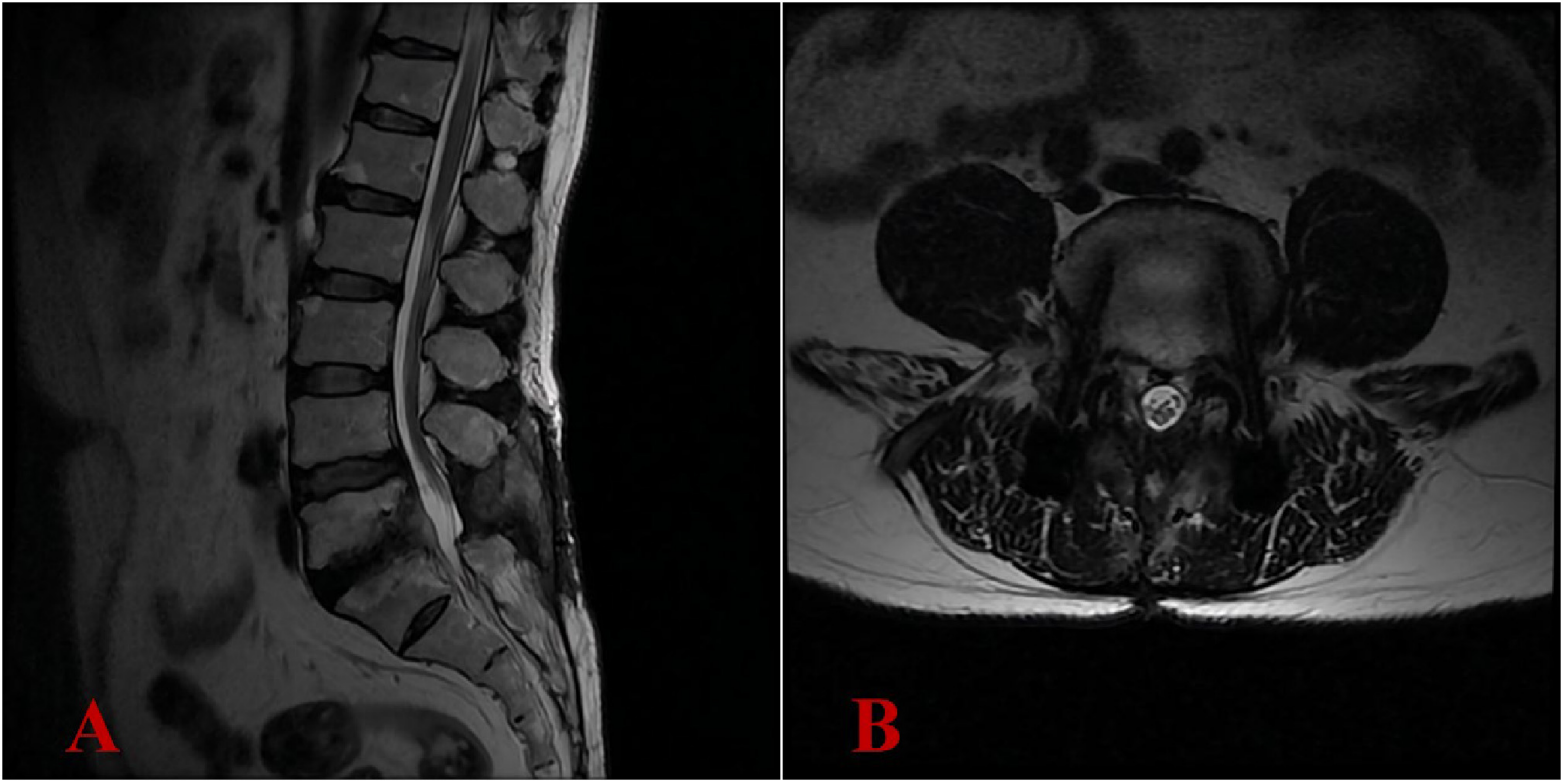
Six-month postoperative lumbar MRI demonstrated complete resolution of the intraspinal abscess, with no recurrence of infection. **(A)** Lateral view; **(B)** transverse view.

At 3 months postoperatively, the ODI showed significant improvement, scoring 15/100 (mild disability), while the VAS for pain intensity demonstrated minimal residual discomfort at 3/10. Results from complete blood count, liver and kidney function tests, ESR, PCT, and CRP were within normal limits. At 6 months postoperatively, the ODI demonstrated exceptional functional recovery, achieving a score of 10/100 (equivalent to normal function), while the VAS for pain intensity exhibited minimal residual discomfort at 1/10 (nearly pain-free). The patient’s clinical symptoms had completely relieved. The lumbar MRI demonstrated complete resolution of the intraspinal abscess, with no recurrence of infection (Figures 5A, B). The antibiotic regimen of oral doxycycline at 200 mg once daily and ciprofloxacin at 1,000 mg once daily was continued, with the plan to maintain anti-infective treatment until 18 months post-surgery (Supplementary Table 1).

## Literature review

A literature review of all reported cases of spinal *Coxiella burnetii* infection spanning the recent 40 years was performed. Two authors independently selected articles related to spinal *Coxiella burnetii* infection from Medline, EMBASE, and Web of Science up to October 2024. Relevant data were extracted from selected articles.

The incidence of spinal *Coxiella burnetii* infection is exceedingly rare, with only a small number of cases reported to date. A synthesis of the reported cases of spinal *Coxiella burnetii* infection highlights the following features (Supplementary Table 2): (1) A predominant number of patients were male (36/42), with an average age of 64 ± 16.89 years; (2) The primary symptom reported was lower back pain (39/42), with fever being the second most common (17/42); (3) Excluding four cases with incomplete records on the detailed location of vertebral infection, The L3 vertebra was most frequently impacted (19/38), followed by L4 (14/38) and L5 (11/38), while the most commonly involved segments were L2-L3, followed by L3-L4, L5-S1, and L4-L5; (4) The mean duration from the onset of symptoms to a confirmed diagnosis was 10.55 months; (5) In terms of therapeutic approaches, for patients exhibiting milder symptoms without abscess or with only partial psoas abscess, anti-infective therapy using hydroxychloroquine or ciprofloxacin in combination with doxycycline was employed; in cases where symptoms did not resolve, or there were significant abscesses or spinal canal abscesses, surgical debridement was critical, followed by prolonged doxycycline therapy for at least 18 months; (6) Excluding the four cases lacking follow-up records, 86.84% (33/38) achieved clinical remission, with the success rate of conservative medical treatment being 80.95%, markedly lower than the 94.12% success rate associated with surgical interventions; (7) In the entire cohort, 59.52% of the patients had vascular involvement. (8) All the cases were diagnosed by serological assays and/or molecular biological testing.

## Discussion

Spinal *Coxiella burnetii* infections affecting the lumbar vertebra are an extremely rare manifestation of chronic Q fever, poses significant diagnostic and therapeutic challenges (17).

### Diagnostic challenges

*Coxiella burnetii* infection demonstrates indistinct inflammatory biomarker profiles, with conventional markers (e.g., CRP, ESR) showing moderate elevations or remaining within normal limits, and *Coxiella burnetii* does not grow in routine cultures using agar-based media (18). Radiological presentation of lumbar *Coxiella burnetii* infection closely resembles that of general pyogenic osteomyelitis. The non-specific nature of clinical symptoms, laboratory findings, and imaging results creates significant diagnostic challenges for lumbar *Coxiella burnetii* infection (19). For most cases, the diagnostic process for lumbar *Coxiella burnetii* infection tends to be protracted and complex (20). Nonetheless, advancements in diagnostic techniques have considerably enhanced the efficiency of diagnosing such infections.

The diagnosis of Q fever necessitates the use of a combination of serological assays, isolation culture, and polymerase chain reaction (PCR) testing (21). Serological tests allow for the differentiation between acute and chronic forms of the disease. Acute Q fever diagnosis can be confirmed by antibodies against phase II antigen of *Coxiella burnetii* are detected earlier and at higher levels compared to the antibodies against phase I antigen; alternatively, a ≥ 4-fold increase in phase II immunoglobulin G (IgG) (22). A phase II: I IgG titer ratio > 1 indicates acute infection, whereas a ratio < 1 correlates with chronic disease, particularly when the phase I IgG titers ≥ 1:800 (23). Nevertheless, serological assays offer only indirect evidence of infection, with false-negative results rates necessitating repeated testing - especially in chronic Q fever where empirical antibiotic therapy and immune tolerance obscure pathogen detection (19). Culture isolation provides definitive evidence of infection but requires Biosafety Level 3 (BSL-3) laboratory, presenting logistical challenges due to technical complexity, high infection risk, and low diagnostic yield - resulting in limited clinical utilization (24). PCR-based testing of blood/tissue specimens enables early diagnosis, as was demonstrated by this case study. Specific multiplex qPCR pathogenic microorganism detection was performed on intraoperative specimens, successfully identifying *Coxiella burnetii*. Metagenomic next-generation sequencing (mNGS) is increasingly employed in infectious diseases diagnostics, demonstrating sensitivity comparable to specific PCR assays and detecting a broader range of pathogens than conventional methods (25). This approach has shown promising utility in detecting fastidious pathogens like *Coxiella burnetii* (26).

### Therapeutic challenges

The treatment of chronic Q fever poses considerable challenges due to *Coxiella burnetii*’s resistance to various antibiotics (27). Doxycycline is regarded as the most efficacious therapeutic option (28–30). Studies revealed that pharmacological management for chronic Q fever does not vary according to the specific sites of infection. Dynamic monitoring of serum antibody titers constitutes a critical component of treatment protocols, necessitating individualized therapy duration adjustments based on serological response kinetics (31, 32). The British Society for Antimicrobial Chemotherapy recommends a combination regimen of hydroxychloroquine or ciprofloxacin with doxycycline for a minimum of 18 months (33). *Coxiella burnetii* resides within the phagolysosome of infected cells, an acidic environment that diminishes doxycycline’s bactericidal activity (34). Hydroxychloroquine incorporation elevates the pH levels within the phagolysosome, thereby selectively enhancing doxycycline’s bactericidal effectiveness (35, 36). Chronic Q fever is considered cured only when IgG phase I antibody titers decline to <1:800 and IgM/IgA phase I antibodies are undetectable (<1:50). Some studies even advocate for lifelong medication in certain cases (37–39). In our case, the patient was found to be in complete symptom remission with no signs of recurrence in imaging at the 6-month postoperative follow-up, so it was questionable whether lifelong medication was required; however, in order to reduce the risk of recurrence, we planned to prolong combination therapy with oral doxycycline and ciprofloxacin until 18 months post-surgery.

This case presents several limitations: (1) we failed to identify this rare lumbar infection prior to the operation. The diagnosis was confirmed until intraoperative specimens detection by multiplex qPCR, and no direct macroscopic visual images of the infection were obtained and preserved during the surgical procedure; (2) the institution did not possess the technical capacity for *Coxiella burnetii* antibody detection, lacked the capability to detect *Coxiella burnetii* antibodies, thus precluding the provision of serological diagnostic evidence. However, specific multiplex qPCR pathogenic microorganism detection was able to provide definitive diagnostic confirmation; (3) as a single case, it lacks broad representativeness, and the follow-up period was limited to only 6 months. Further cases with extended follow-up periods are necessary to verify the efficacy of the treatment protocol.

## Conclusion

The combined approach of decompression through total laminectomy, lesion excision, intervertebral bone graft fusion, and fixation, supplemented with oral doxycycline and ciprofloxacin therapy, was demonstrated to be an effective treatment strategy for *Coxiella burnetii* infection in the lumbar vertebra.

## Data Availability

The original contributions presented in this study are included in this article/Supplementary material, further inquiries can be directed to the corresponding author.
