# Supplementary material for: Coxiella burnetii infection in the lumbar vertebra: a rare case report and review of literature
Source: Front Med (Lausanne). 2025 Aug 1;12:1618670. doi: 10.3389/fmed.2025.1618670 (PMC12354406; doi:10.3389/fmed.2025.1618670)
Supplement: Supplementary file 1 [file Table_1.doc]

**Table 1: Clinical Timeline of Patient Care**

| **Date** | **Event** | **Key Clinical Details & Outcomes** |
| --- | --- | --- |
| 20/12/2023 | Initial presentation | Worsening LBP (2 months) + bilateral lower limb pain (2 weeks). MRI: L5/S1 infection (Fig 1AB) |
| 20/12/2023 | First intervention | Percutaneous endoscopic discectomy. Culture: negative. Histopathology: chronic granulomatous inflammation |
| 20/12/2023 - 25/04/2024 | Empirical therapy | 5-month anti-TB regimen (no improvement in VAS/ODI scores) |
| **25/04/2024** | **Hospital admission** | VAS: 6/10, ODI: 62/100. CT: L5/S1 bone destruction (Fig 2AB). MRI: epidural abscess (Fig 2CD) |
| **26/04/2024** | **Surgical intervention** | L5-S1 laminectomy + debridement + fusion + fixation. Intraoperative: purulent exudate observed |
| **27/04/2024** | **Diagnostic confirmation** | qPCR positive for C. burnetii (Ct=29.24). Antibiotics initiated: doxycycline 200mg + ciprofloxacin 1000mg daily |
| 27/07/2024 | 3-month follow-up | VAS: 3/10 (-50%), ODI: 15/100 (-76%). MRI: resolving abscess. Lab: normal CRP/ESR |
| **27/10/2024** | **6-month follow-up** | **VAS: 1/10, ODI: 10/100.** MRI: no recurrence (Fig 5AB). Full symptom resolution |
| Planned (2026) | Treatment completion | 18-month antibiotic course (ongoing) |
