# Supplementary material for: Coxiella burnetii infection in the lumbar vertebra: a rare case report and review of literature
Source: Front Med (Lausanne). 2025 Aug 1;12:1618670. doi: 10.3389/fmed.2025.1618670 (PMC12354406; doi:10.3389/fmed.2025.1618670)
Supplement: Supplementary file 2 [file Table_2.docx]

Table 2. All reported cases of spinal *Coxiella burnetii* infection spanning the recent 40 years.

|  | | | | | | | | | | | | | | | |
| --- | --- | --- | --- | --- | --- | --- | --- | --- | --- | --- | --- | --- | --- | --- | --- |
| surgical intervention | | | | | | | | | | | | | | | |
| Case Number | **Year** | **With vascular infection** | **Duration（months）** | **Age (years)/ Gender** | **Level** | **Paravertebral abscess** | **epidural abscess** | **Clinical symptoms** | **Exposure risk factors** | **Diagnosis Method** | **surgical strategy** | **Antibiotic regimen** | **Treatment duration（months）** | **Follow-up（months）** | **Outcomes** |
| Piquet^[1]^ | **1994** | Y | 4 | 67/male | L2 | N | N | lumbar pain/weight loss | Y | PCR/serology | Drainage of paravertebral abscesses, debridement of infected tissues | DOX+OFL | 36 | 36 | Cured, Well at 36 months postsurgery |
| Heldenberg^[2]^ | **2015** | Y | 24 | 54/male | L3-L4 | Y | N | back pain | N | serology | Abscess evacuation | DOX+HCQ+RIF+MET | plan lifelong | NA | NA |
| Galy^[3]^ | **2016** | Y | 36 | 92/male | L3 | Y | Y | back pain | N | serology | Abscess evacuation | DOX+HCQ | plan 18 | 6 | Well at 6 months postsurgery |
| Jayet^[4]^ | **2016** | Y | NA | 91/male | L3 | Y | Y | back pain/fever | N | serology | Drainage of paravertebral abscesses, debridement of infected tissues | DOX+HCQ | 18 | 20 | Cured ,Well at 20 months postsurgery |
| Leahey ^[5]^ | **2016** | Y | 8 | 57/male | L3-L4 | Y | Y | back pain/fever | Y | PCR/serology | Decompression with laminectomy and fusion; | DOX+HCQ | plan lifelong | 3 | Cured ,Well at 3 months postsurgery |
| Virk^[6]^ | **2017** | Y | 6 | 67/male | L2-L3 | Y | N | back pain/ sweats/malaise | Y | PCR | Discectomy and retroperitoneal debridement | DOX+HCQ | plan 24 | 6 | Well 6 at months postsurgery |
| Virk^[6]^ | **2017** | Y | NA | 57/ female | L2-L4 | Y | N | Asymptomatic—noted on staging CT for  cancer | Y | PCR | L2-L4 and retroperitoneal debridement | DOX+CIP | NA | 72 | Cured, Died at 72 months—metastatic colon cancer |
| Virk^[6]^ | **2017** | Y | 3 | 74/male | L1-L3 | Y | Y | back pain | Y | PCR | Open debridement | DOX+HCQ | plan 12–18 | 1.8 | Died of secondary complications |
| Virk^[6]^ | **2017** | Y | NA | 60/ female | L2-L4 | Y | N | back pain/ weight loss/ gait instability | Y | PCR | The spine debridement and stabilization with hardware fixation PLIF | DOX+CIP | NA | 78 | Cured, Well at 78 months postsurgery |
| Mongardon^[7]^ | **2018** | Y | 2 | 66/male | L2-L3 | Y | N | back pain/fever | Y | serology | drained the abscesses and osteosynthesis of the lumbar spine | DOX+HCQ | NA | 6 | Well at 6 months postsurgery |
| Lundy^[8]^ | **2019** | Y | 5 | 74/male | L5-S1 | Y | N | back pain/ radiculopathy | N | PCR | Discectomy and retroperitoneal washout | DOX+HCQ | plan 12–18 | 3 | Well at 3 months postsurgery |
| Waelbers^[9]^ | **2020** | Y | Acute | 69/ female | L3-L5 | Y | Y | back pain/fever | Y | PCR/serology | Open biopsy and Drainage of paravertebral abscessest | DOX+HCQ | plan 18 | 5 | Cured, Well at 5 months postsurgery |
| Dorfman^[10]^ | **2021** | N | 2 | 78/female | T11-T12 | Y | N | epigastric pain/weight loss/anorexi | N | PCR/serology | Percutaneously drained continuously  for 8 months | DOX+HCQ | plan 18 | 1 | Well at 1 month postsurgery |
| Ghanem-Zoubi^[11]^ | **2021** | N | 2 | 79/female | T10-L1 | N | N | fever/weight loss/right upper abdominal pain | N | serology | CT guided drain insertion yielded | DOX+HCQ | plan 18 | 12 | Well at 1 month postsurgery，but phase 1 serology persists |
| Ghanem-Zoubi^[11]^ | **2021** | Y | 2 | 66/male | L5 | Y | N | back pain/weight loss | N | serology | A percutaneous puncture of the psoas abscess yielded a small amount of cloudy fluid. | DOX+HCQ+LEVO | plan 18- 24 | 6 | Well at 6 months postsurgery |
| Yang^[12]^ | **2023** | N | 36 | 66/male | L1-L2 | N | Y | low back pain/numbness /lower limbs weakness | Y | mNGS testing | anterior debridement and fusion plus posterior fusion with instrumentation | DOX +RIF+LEVO | plan 18 | 16 | Well at 16 months postsurgery |
| Mangkalamanee^[13]^ | **2024** | N | 12 | 64/male | L3-S1 | Y | Y | back pain | Y | PCR | Decompressive laminectomy with pedicle fusion | DOX+HCQ+RIF | NA | 2 | Well at 2 months postsurgery |
| Current case | **2024** | N | 6 | 57/male | L5-S1 | Y | Y | back pain/ sweats/malaise /weight loss | Y | PCR | PLIF | DOX+CIP | NA | 3 | Cured, Well at 3 months postsurgery |
| conservative therapy | | | | | | | | | | | | | | | |
| Ellis^[14]^ | **1983** | N | NA | 76/female | T12/L1 | Y | Y | back pain/fever | Y | serology | NA | RIF+ISO+ETH | 24 | 24 | NA |
| Ellis^[14]^ | **1983** | N | 12 | 39/male | L5 | N | N | back pain/fever/ weight loss | Y | serology | NA | TET | 5 | 5 | Cured, Good prognosis at 5 months |
| Cottalorda^[15]^ | **1995** | N | 8 | 7/male | L3 | N | N | back pain/fever | Y | serology/ culture | NA | DOX+RIF | DOX24/RIF6 | 24 | Cured, Good prognosis at 24 months |
| Fournier^[16]^ | **1998** | Y | NA | 70/male | NA | N | N | back pain/fever | Y | serology/ culture | NA | DOX | plan 18 | 2 | Died postoperatively— aortoduodenal fistula |
| Fournier^[16]^ | **1998** | Y | NA | 70/male | NA | N | N | fever | N | serology/ culture | NA | DOX | 18 | 18 | Cured, Good prognosis at 18 months |
| Nourse^[17]^ | **2004** | N | 3 | 4/male | T10 | N | N | back pain/fever | Y | PCR/serology | NA | DOX+HCQ | 36 | 36 | Cured, Good prognosis at 336 months |
| Costa^[18]^ | **2006** | N | NA | 67/male | NA | N | N | back pain/fever | N | serology | NA | DOX | NA | NA | Cured |
| Breton^[19]^ | **2007** | Y | 2 | 66/male | L3-L4 | Y | N | lumbar pain/ feve | Y | PCR/serology | NA | DOX+HCQ | 36 | 36 | Cured, Good prognosis at 36 months |
| Breton^[19]^ | **2007** | Y | NA | 65/male | L4-L5 | Y | N | back pain/weight loss | N | PCR/serology | NA | DOX+HCQ+OFL | plan 18 | NA | NA |
| Landais^[20]^ | **2007** | N | 2 | 47/male | L5-S1 | N | Y | back pain/ radiculopathy | N | PCR/serology | NA | DOX+HCQ | NA | 12 | Cured ,Good prognosis at 12 months |
| Landais^[20]^ | **2007** | Y | 1 | 64/male | L2-L3/ L4-L5 | Y | Y | back pain/fever | N | PCR/serology/ culture | NA | DOX+HCQ+RIF | NA | 12 | Good prognosis at 12 months |
| O'Donnell^[21]^ | **2007** | Y | NA | 66/male | L1-L3 | Y | N | Back pain/fever | Y | serology | NA | DOX+CIP | plan lifelong | 21 | Good prognosis at 21 months |
| Merhej^[22]^ | **2012** | Y | 26 | 70/male | L4 | Y | N | back pain/weight loss | Y | PCR/ serology | NA | DOX+HCQ | plan 18 | 6 | Improved titers at 6 months |
| Aerts^[23]^ | **2013** | Y | 24 | 77/male | NA | Y | N | back pain | N | PCR/ serology | NA | DOX+HCQ | NA | NA | Died postoperatively— ischemic colon |
| Gaudé^[24]^ | **2016** | N | NA | 55/male | L4-L5 | Y | N | back pain | Y | serology | NA | DOX+HCQ | 10 | 10 | Good prognosis at 10 months |
| Michel^[25]^ | **2016** | Y | 9 | 64/male | L3-L4 | Y | N | back pain/malaise | N | PCR/serology | NA | DOX+HCQ | NA | NA | NA |
| Stokes^[26]^ | **2016** | Y | 48 | 67/male | L1-L3 | Y | N | back & abdominal pain | N | PCR | NA | DOX+HCQ | NA | 18 | Died at 18 months— pulmonary embolism |
| Meriglier^[27]^ | **2018** | N | NA | 86/male | L2-L3 | Y | N | back pain/weakness | N | PCR | NA | DOX+HCQ | 18 | 18 | good prognosis |
| Meriglier^[27]^ | **2018** | N | NA | 66/male | L4-S1 | Y | N | back pain/ radiculopathy | N | PCR/serology | NA | DOX+HCQ | 18 | 18 | good prognosis |
| Meriglier^[27]^ | **2018** | N | NA | 59/male | L5-S1 | Y | Y | back pain | N | PCR/serology | NA | DOX+HCQ | 18 | 18 | good prognosis |
| Browning^[28]^ | **2020** | N | 4 | 58/male | L5-S1 | Y | N | back pain/fever | N | qPCR/serology | NA | DOX+HCQ | plan 18 | 12 | Good prognosis at 12 months |
| Browning^[28]^ | **2020** | N | 6 | 55/male | T8-T11 | Y | Y | back pain/ weight loss/ gait instability | Y | PCR/serology | NA | MET+SUL | 20 | 32 | Good prognosis at 20 months /Improved titers at 32 months |
| Ghanem-Zoubi^[11]^ | **2021** | Y | NA | 72/male | L3 | N | N | back pain | N | serology | NA | DOX+HCQ | NA | 18 | Good prognosis at 18 months, But phase 1 IgG still 1600 |
| Ghanem-Zoubi^[11]^ | **2021** | Y | 2 | 80/male | L4 | Y | N | back pain/fever /weight loss | Y | serology | NA | DOX+HCQ+MOXI | plan 24 | 18 | Good prognosis at 18 months |
|  |  |  |  |  |  |  |  |  |  |  |  |  |  |  |  |
| DOX doxycycline, HCQ hydroxychloroquine, RIF rifampicin, CIP ciprofloxacin, CLIN clindamycin, TET tetracycline, ISO isoniazid, ETH ethambutol, OFL ofloxacine, MET metronidazole, SUL sulfamethoxazole, LEVO levofloxacin, MOXI moxifloxacin | | | | | | | | | | | | | | | |

1. Piquet, P, Raoult D, Tranier P, Mercier C. *Coxiella burnetii* infection of pseudoaneurysm of an aortic bypass graft with contiguous vertebral osteomyelitis. *J Vasc Surg*. (1994) 19:165–8. doi: 10.1016/s0741-5214(94)70131-8

2. Heldenberg E, Rabin I, Peer A, Karplus R, Bass AA. Creative approach to mycotic abdominal aortic aneurysm secondary to *Coxiella burnetii* Infection. *Isr Med Assoc J*. (2015) 17:385–6. PMID : 26234001.

3. Galy A, Decousser J, El-Anbassi S, Nebbad B, Belzunce C, Cochennec F, et al. Psoas abscess and chronic Q fever: a contiguous or hematogenous complication? A case report and literature review. *Infect Dis*. (2016) 48:626–31. doi: 10.1080/23744235. 2016.1180709.

4. Jayet J, Raux M, Allaire E, Desgranges P, Cochennec F. Treatment of an abdominal aortic aneurysm infected by *Coxiella Burnetii* using a cryopreserved allograft. *Ann Vasc Surg*. (2016) 33:227.e9–e12. doi: 10.1016/j.avsg.2015.09.031.

5. Leahey P, Tahan S, Kasper E, Albrecht M. Chronic Q-Fever (Coxiella burnetii) causing abdominal aortic aneurysm and lumbar osteomyelitis: a case report. Open Forum Infect Dis. (2016) 3:ofv185. doi: 10.1093/ofid/ofv185.

6. Virk A, Mahmood M, Kalra M, Bower T, Osmon D, Berbari E, et al. *Coxiella burnetii* multilevel disk space infection, epidural abscess, and vertebral osteomyelitis secondary to contiguous spread from infected abdominal aortic aneurysm or graft: report of 4 cases acquired in the US and review of the literature. *Open Forum Infect Dis*. (2017) 4:ofx192. doi: 10.1093/ofid/ofx192.

7. Mongardon N, Dubory A, Dorget A, De Wailly G, Lepeule R, Cochennec F. Vascular infection and vertebral destruction in a patient with Q fever. *Lancet Infect Dis*. (2018) 18:226. doi: 10.1016/S1473-3099(17)30477-2.

8. Lundy P, Arnold P, Hance K. Coxiella burnetii infection of the spine requiring neurosurgical intervention. *Surg Neurol Int*. (2019) 10:182. doi: 10.25259/SNI_205_2019.

9. Waelbers V, Desmet S, De Munter P, Van Loon J, Fourneau I. Vertebral osteomyelitis or infected abdominal aortic endograft? A rare case of Q Fever. *Ann Vasc Surg*. (2020) 67:568.e9–e12. doi: 10.1016/j.avsg.2020.03.018.

10. Dorfman K, Eran A, Ghanem-Zoubi NQ. Fever vertebral osteomyelitis complicating vertebroplasty. *Rambam Maimonides Med J*. (2021) 12:e0007. doi: 10. 5041/RMMJ.10430

11. Ghanem-Zoubi N, Karram T, Kagna O, Merhav G, Keidar Z, Paul M. Q fever vertebral osteomyelitis among adults: a case series and literature review. *Infect Dis*. (2021) 53:231–40. doi: 10.1080/23744235.2020.1871508.

12. Yang S, Xue B, Hu X, Zhou W, Zhang M, Zhao M. Spinal infection caused by *Coxiella burnetii*. *BMC Infect Dis*. (2023) 23:6. doi: 10.1186/s12879-022-07938-7.

13. Mangkalamanee O, Rotcheewaphan S, Phuensan P, Ponpinit T, Hemachudha T, Torvorapanit P. The first human case report of molecularly confirmed co-infection of *Brucella melitensis* and *Coxiella burnetii*: a case report. *Heliyon*. (2024) 10:e29685. doi: 10.1016/j.heliyon.2024.e29685.

14. Ellis M, Smith C, Moffat M. Chronic or fatal Q-fever infection: a review of 16 patients seen in North-East Scotland (1967-80). *Q J Med*. (1983) 52:54–66. doi: 10.1093/oxfordjournals.qjmed.a067742.

15. Cottalorda J, Jouve J, Bollini G, Touzet P, Poujol A, Kelberine F, et al. Osteoarticular infection due to *Coxiella burnetii* in children. *J Pediatr Orthop B*. (1995) 4:219–21. doi: 10.1097/01202412-199504020-00018.

16. Fournier P, Casalta J, Piquet P, Tournigand P, Branchereau A, Raoult D. *Coxiella burnetii* infection of aneurysms or vascular grafts: report of seven cases and review. *Clin Infect Dis*. (1998) 26:116–21. doi: 10.1086/516255.

17. Nourse C, Allworth A, Jones A, Horvath R, McCormack J, Bartlett J, et al. Three cases of Q fever osteomyelitis in children and a review of the literature. *Clin Infect Dis*. (2004) 39:e61–6. doi: 10.1086/424014.

18. Costa P, Brigatte M, Greco D. Questing one Brazilian query: reporting 16 cases of Q fever from Minas Gerais, Brazil. *Rev Inst Med Trop Sao Paulo*. (2006) 48:5–9. doi: 10.1590/s0036-46652006000100002.

19. Breton G, Yahiaoui Y, Deforges L, Lebrun A, Michel M, Godeau B. Psoas abscess: an unusual manifestation of Q fever. *Eur J Intern Med*. (2007) 18:66–8. doi: 10.1016/j. ejim.2006.07.018.

20. Landais C, Fenollar F, Constantin A, Cazorla C, Guilyardi C, Lepidi H, et al. Q fever osteoarticular infection: four new cases and a review of the literature. *Eur J Clin Microbiol Infect Dis*. (2007) 26:341–7. doi: 10.1007/s10096-007-0285-5.

21. O’Donnell M, Manshani N, McCaughey C, Soong C, Lee B. *Coxiella burnetii* infection of an aortic graft with multiple vertebral body erosion. *J Vasc Surg*. (2007) 45:399–403. doi: 10.1016/j.jvs.2006.09.016.

22. Merhej V, Cammilleri S, Piquet P, Casalta J, Raoult D. Relevance of the positron emission tomography in the diagnosis of vascular graft infection with *Coxiella burnetii*. *Comp Immunol Microbiol Infect Dis*. (2012) 35:45–9. doi: 10.1016/j.cimid.2011. 09.010.

23. Aerts P, van Zitteren M, Van Kasteren M, Buiting A, Heyligers J, Vriens P. Report of two in situ reconstructions with a saphenous spiral vein graft of *Coxiella burnetii*-infected aneurysms of the abdominal aorta. *J Vasc Surg*. (2013) 57:234–7. doi: 10.1016/j.jvs.2012.08.042.

24. Gaudé M, Julien S, Laurent F, Ferry T. Disappearance of FDG uptake on PET scan after antimicrobial therapy could help for the diagnosis of *Coxiella burnetii* spondylodiscitis. *BMJ Case Rep*. (2016) 2016:bcr2015214008. doi: 10.1136/bcr-2015-214008.

25. Michel M, Cesini J, Michon J, Dargere S, Vergnaud M, Marcelli C. Vertebral fractures and abdominal aortic aneurysm revealing Q fever. *Joint Bone Spine*. (2016) 83:241–2. doi: 10.1016/j.jbspin.2015.08.001.

26. Stokes W, Janvier J, Vaughan S. Chronic Q Fever in alberta: a case of *Coxiella burnetii* mycotic aneurysm and concomitant vertebral osteomyelitis. *Can J Infect Dis Med Microbiol*. (2016) 2016:7456157. doi: 10.1155/2016/7456157.

27. Meriglier E, Sunder A, Elsendoorn A, Canoui E, Rammaert B, Million M, et al. Osteoarticular manifestations of Q fever: a case series and literature review. *Clin Microbiol Infect*. (2018) 24:912–3. doi: 10.1016/j.cmi.2018.03.005.

28. Browning S, Lai K, Pickles R, Graves SR. Q fever vertebral osteomyelitis in the absence of cardiovascular involvement: two cases and a literature review. *Clin Infect Pract*. (2020) 6:100019. doi: 10.1016/j.clinpr.2020.100019.
